# Iron Therapy in Pediatric Iron Deficiency and Iron-Deficiency Anemia: Efficacy, Safety, and Formulation-Specific Trade-Offs—A Narrative Review

**DOI:** 10.3390/hematolrep18010006

**Published:** 2026-01-03

**Authors:** Guido Leone, Marta Arrabito, Giovanna Russo, Milena La Spina

**Affiliations:** 1 Department of Clinical and Experimental Medicine, University of Catania, 95123 Catania, Italy; guido.leone@outlook.com; 2 Azienda Policlinico “Rodolico-San Marco”, 95123 Catania, Italy; marta-arrabito@hotmail.it (M.A.); mlaspina@unict.it (M.L.S.)

**Keywords:** children, infants, iron-deficiency anemia, ferrous sulfate, ferrous bisglycinate, liposomal iron, sucrosomial iron, hepcidin, tolerability, intravenous iron

## Abstract

**Background/Objectives**: Iron deficiency (ID) is the most common nutritional disorder in childhood worldwide. It has profound consequences for growth, neurodevelopment, behaviour, and overall health. Despite the long-standing efficacy of oral ferrous salts, their poor gastrointestinal tolerability and adherence challenges have spurred the development of alternative formulations and innovative dosing strategies. **Methods**: We conducted a narrative review of national and international guidelines, pediatric randomized controlled trials, observational and cohort studies, cost-effectiveness analyses, diagnostic method papers, and reviews, with emphasis on diagnostic innovations, therapeutic outcomes, tolerability, and formulation-specific efficacy. **Results**: Ferrous salts remain the gold standard for efficacy, low cost, and guideline endorsement, but up to 40% of children experience GI intolerance. Therefore, a lower dosage of ferrous salts has been proposed for IDA as still being an efficacious and better-tolerated schedule. Also, alternate-day dosing improves absorption and tolerability and is supported by a recent pediatric RCT. Newer formulations—ferric polymaltose, ferrous bisglycinate, co-processed bisglycinate with alginate (Feralgine™), and vesicular encapsulated forms such as sucrosomial and liposomal ferric pyrophosphate—showed improved tolerability and palatability, supporting adherence with hematologic outcomes comparable to ferrous salts, particularly in children with intolerance, malabsorption, or inflammatory comorbidities. Intravenous iron is effective and safe with modern preparations and is reserved for severe anemia, malabsorption, or oral therapy failure. **Conclusions**: Oral ferrous salts should remain the first-line therapy in pediatric ID/IDA. Future pediatric trials should prioritize head-to-head comparisons of formulations, hepcidin-guided dosing, and patient-centred outcomes, including neurocognitive trajectories and quality of life.

## 1. Introduction

Iron deficiency (ID), with or without anemia (iron-deficiency anemia, IDA), is the most frequent nutritional disorder in childhood worldwide. Its prevalence peaks in two critical pediatric windows: late infancy/toddlerhood, when rapid somatic and neurodevelopmental growth markedly increases iron requirements, and adolescence, particularly in females due to menstrual losses [1]. Beyond its hematologic manifestations, ID has broad systemic consequences, including impaired cognitive development, behavioural alterations, reduced physical capacity, and compromised immune function. If left untreated, IDA in early life may cause long-lasting neurodevelopmental deficits that persist despite later correction of hematological parameters [2,3]. The etiology of pediatric IDA is multifactorial. Nutritional inadequacy is common during the transition from exclusive breastfeeding to complementary feeding, when dietary iron content is often inadequate. Excessive intake of cow milk, prevalent in toddlers, further limits dietary iron and may contribute to occult blood loss when cow milk protein allergy coexists. In older children and adolescents, menorrhagia, gastrointestinal disease (celiac disease, inflammatory bowel disease, Helicobacter pylori infection, chronic autoimmune gastritis, etc.), and dietary restrictions (vegan/vegetarian diet) frequently contribute to ID and IDA [4]. Infection and chronic inflammation amplify the problem by elevating hepcidin levels, thereby suppressing ferroportin-mediated iron absorption and recycling [4,5,6]. The therapeutic armamentarium for pediatric ID/IDA is dominated by oral iron, traditionally ferrous salts such as sulfate, fumarate, or gluconate [7]. These are effective, unexpensive, and universally recommended as first-line therapy, but their use is limited by gastrointestinal intolerance, poor palatability, and adherence issues. Alternative preparations, including ferric polymaltose, iron polysaccharide complexes, amino-acid chelates (ferrous bisglycinate), and newer encapsulated formulations such as liposomal or sucrosomial ferric pyrophosphate, have been developed to overcome these limitations. Each compound possesses distinct pharmacokinetic properties, efficacy profiles, and tolerability trade-offs, which are particularly important in pediatrics, where adherence, palatability, and safety are paramount. Moreover, while intravenous (IV) iron has proven safe and effective in selected pediatric populations, particularly in IBD, celiac disease, or severe refractory anemia, its role relative to optimized oral strategies requires further clarification [2,5].

This narrative review reports current evidence on the efficacy, safety, and tolerability of iron therapy in children with ID and IDA, with particular attention to formulation-specific advantages and drawbacks. It integrates international guidelines, pediatric randomized and observational studies, diagnostic innovations, observational and cohort studies, reviews, and selected adult data when pediatric evidence is lacking. This review also considers neurodevelopmental and growth outcomes, practical aspects of dosing, pitfalls in management, and health-system implications, aiming to provide clinicians with a comprehensive, formulation-aware framework for the management of pediatric ID and IDA.

## 2. Materials and Methods

We searched and synthesized evidence from multiple sources relevant to pediatric iron deficiency (ID) and iron-deficiency anemia (IDA), including international and national clinical guidelines, randomized controlled trials, observational and cohort studies, cost-effectiveness analyses, and diagnostic method papers. The main databases consulted were PubMed/MEDLINE and Embase, complemented by manual searches of reference lists and grey literature. Studies were not restricted by publication year, but priority was given to the recent high-quality pediatric literature. When pediatric-specific evidence was limited or absent, carefully selected adult data were considered, with extrapolation explicitly labelled as such.

Data extraction focused on the following: diagnostic approaches (complete blood count, sideremia, ferritin, transferrin saturation, reticulocyte hemoglobin content, hepcidin, soluble transferrin receptor), treatment outcomes (hemoglobin, ferritin, transferrin saturation, reticulocyte indices), neurodevelopmental and growth endpoints, formulation-specific efficacy and safety, and indications for intravenous iron. Special emphasis was placed on tolerability, adherence, and practical implementation in routine pediatric care.

As this is a narrative review, no formal risk-of-bias assessment or quantitative synthesis (meta-analysis) was performed. Instead, findings were integrated thematically and critically appraised to generate a comprehensive overview of current knowledge, highlight areas of consensus and controversy, and identify gaps requiring further research.

## 3. Results

### 3.1. Diagnosis

Mild to moderate iron deficiency in the absence of anemia may be clinically silent, although some children can present with nonspecific symptoms such as fatigue or reduced exercise tolerance [8]. When iron deficiency progresses to moderate or severe stages and is accompanied by anemia, the clinical picture typically includes pallor, asthenia, and other hallmark features of impaired erythropoiesis. Additional manifestations may encompass restless legs syndrome, trophic alterations of the mucosa such as stomatitis and glossitis, pica, and increased susceptibility to infections, as well as neuropsychiatric symptoms including mood disturbances, behavioural changes, and a decline in school performance [4]. Particularly concerning is the evidence that iron deficiency during early infancy is associated with irreversible neurocognitive sequelae, impaired learning potential, and deficits in motor development, underscoring the critical importance of early recognition and intervention [2,3,9,10].

Initial laboratory work-up includes CBC (reduced Hb, microcytosis, hypochromia, elevated RDW), ferritin (low unless inflammation), and other iron indices (low sideremia and transferrin saturation—TSAT) [2,11]. Complementary markers, such as soluble transferrin receptor (sTfR), reticulocyte hemoglobin content (CHr/Ret-He), and percentage hypochromic erythrocytes (%HYPO), can be used to detect functional iron-restricted erythropoiesis when ferritin is unreliable. The ratio of sTfR to ferritin, which is high in iron-deficiency anemia and low in chronic disease anemia, is useful in the differential diagnosis between these two conditions [2,11].

While not universally standardized, in selected cases, serum hepcidin can help differentiate ID/IDA from anemia of inflammation and to gauge absorption potential; pediatric cut-offs remain mainly unsettled. A study reported that hepcidin ≤ 6.895 ng/mL had a sensitivity of 79.2% and specificity of 82.8% for the diagnosis of ID [12]. Pediatric lab-hematology reviews support Ret-He as a fast and functional indicator of iron-restricted erythropoiesis (Ret-He appears low in IDA), while hepcidin reflects the absorptive “gate” and can be low in pure ID and high in inflammatory states [6].

**Figure 1:**
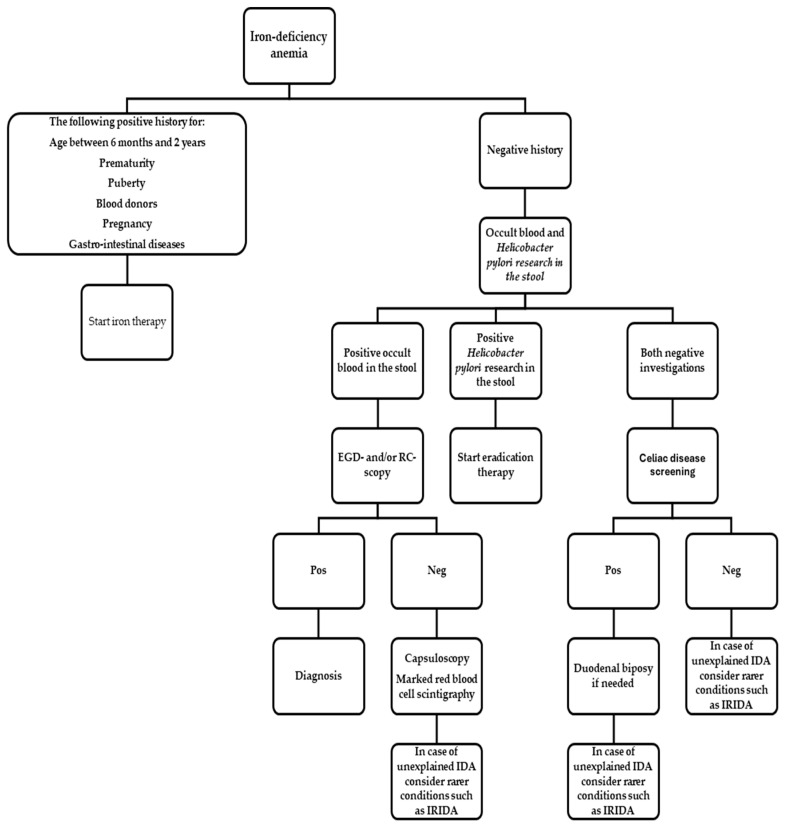
Diagnostic approach for IDA. EGD-scopy, esophagogastroduodenoscopy; RC-scopy, rectocolonscopy; IDA, iron-deficiency anemia; IRIDA, iron-refractory iron-deficiency anemia.

As regards the etiology of ID/IDA, a thorough dietary and bleeding history, birth and growth review, and assessment of chronic disease/inflammation should accompany laboratory work-up. Patients are initially stratified based on the presence or absence of a positive history for specific risk factors, including age between 6 months and 2 years, prematurity, puberty, fertile age, blood donation, pregnancy, gastro-intestinal disease. Prolonged exclusive breastfeeding beyond 6–12 months can lead to ID, especially when it delays the introduction of iron-rich complementary foods. During adolescence, iron need increases due to factors such as inadequate diet, consumption of iron absorption inhibitors, malnutrition, obesity, menstrual blood loss in females, and iron loss related to sports activity. In adolescents, heavy menstrual bleeding, occasionally due to undiagnosed Von Willebrand disease, should be considered. Prematurity, neuromotor disorders, and GI diseases contribute to IDA. In the presence of one or more of these conditions, iron deficiency is usually temporary, and iron supplementation alone can restore iron content; iron therapy should be initiated with no need for further work-up (Figure 1) [4]. In the absence of a relevant history, identifying the underlying condition is of paramount importance, as only addressing the cause of ID can lead to its potential long-term resolution.

The next step is the assessment of occult blood and the research of Helicobcter pylori in the stool. If occult blood in the stool is positive, EGD and/or RC-scopy should be performed; if the research of Helicobacter pylori is positive, eradication therapy should be started. If these investigations are positive, a diagnosis can be made; if EGD and/or RC-scopy are negative, in presence of positive occult blood in the stool, further evaluation with capsuloscopy or marked red blood cell scintigraphy is suggested. If occult blood and Helicobacter pylori in the stool are negative, celiac disease screening is indicated: if positive, a duodenal biopsy is sometimes required. Finally, in the case of unexplained IDA, rarer conditions such as iron-refractory iron-deficiency anemia (IRIDA) should be considered (Figure 1) [4]. Etiologic identification must go in tandem with appropriate iron replacement therapy rather than waiting, especially when IDA is severe.

### 3.2. Neurodevelopment, Behaviour, and Growth

High-quality clinical trials showed heterogeneous evidence regarding the developmental benefits of routine iron supplementation in otherwise broadly healthy infants. For instance, the large, rigorously conducted BRISC trial in Bangladesh evaluated the effect of three months of daily iron syrup or multiple micronutrient powders during infancy and subsequently assessed neurodevelopmental outcomes at 3 years of age. Despite a demonstrable reduction in the prevalence of anemia, the investigators did not observe improvements in standardized cognitive scores, highlighting that large-scale population supplementation strategies may not necessarily translate into measurable gains in neurocognitive performance in all epidemiological contexts. This underscores a crucial conceptual distinction between untargeted prophylactic supplementation in unselected populations and therapeutic intervention directed to children with confirmed iron deficiency or iron-deficiency anemia (ID/IDA), where the biological rationale and expected benefits are substantially stronger. However, several factors such as infections, socioeconomic and environmental confounders, coexisting nutritional deficiencies, etc. can limit the cognitive benefits of population level iron supplementation [13].

Conversely, more recent pediatric randomized, double-blind studies provided encouraging data with novel formulations. A trial investigating liposomal iron administered over a four-month period demonstrated significant improvements in developmental composite scores in children with non-anemic iron deficiency (NAID) when compared with placebo. These findings are particularly relevant because they indicate that targeted treatment, even prior to the development of overt anemia, may confer measurable neurodevelopmental advantages. Moreover, the trial reported favourable tolerability of liposomal preparations, suggesting that enhanced palatability and reduced gastrointestinal side effects may contribute to consistent adherence and thereby potentiate the observed clinical benefits [14].

The impact of iron supplementation on somatic growth outcomes has been less consistent across the pediatric literature. Several trials and cohort studies have shown no measurable effect on growth parameters, while others have documented modest benefits in children with established IDA. By contrast, some reports have even signalled potential adverse effects, particularly on linear growth, when supplementation is provided to iron-replete infants. This body of evidence reinforces the notion that iron therapy should be judiciously targeted to children with documented deficiency rather than administered indiscriminately, both to maximize benefit and to minimize unintended harm. Furthermore, local epidemiological factors—especially the background prevalence of infections and the interaction between iron supplementation and host–pathogen dynamics—must be carefully considered in interpreting study results and in shaping public health recommendations. Collectively, these observations emphasize the complexity of balancing efficacy, safety, and long-term developmental outcomes in pediatric iron supplementation strategies, and they highlight the critical importance of individualized, evidence-based therapeutic decision-making [3].

### 3.3. Therapeutic Goals, Dosing Strategies, and Pharmacological Considerations

#### 3.3.1. Goals

The immediate goal is symptom relief and hemoglobin correction; the medium-term goal is normalization of red cell indices and replenishment of stores; the long-term objective is relapse and long term sequalae prevention. Pediatric hematology outcome data quantify expected trajectories: with adequate oral therapy and adherence, reticulocytosis appears within a week, hemoglobin typically rises by ~1–2 g/dL within four weeks, and microcytosis and ferritin normalization lag hemoglobin by weeks to months [2,4]. “Time-to-resolution” data in young children validate that microcytosis and iron stores may require several months beyond hemoglobin recovery, highlighting the importance of continuing oral iron 2–3 months after normalization to rebuild stores [15]. Observational pediatric data suggest that time to full resolution scales with baseline severity, underscoring early follow-up and dose optimization [4,6].

#### 3.3.2. Standard Pediatric Dosing

The recommended dosage of iron as standard oral therapy is mainly unstudied, and standard dosing derives from custom or textbook indications and transmitted over the years. Lately, there is a trend toward using lower doses to prevent side effects, since the amount of iron absorbed is constant, and any excess that is not absorbed results in adverse effects. Standard pediatric dosing for each compound, based on textbooks and taken from available evidence, is reported below.

- Ferrous salts: classical pediatric dosing is 2–3 mg/kg/day of elemental Fe^2+^ iron for ID and up to 3–6 mg/kg/day for IDA, delivered once daily or in divided doses (two to three) [2,5].
- Ferric complexes (iron polymaltose/iron polysaccharide): somewhat higher elemental Fe^3+^ iron (i.e., dose at 3–5 mg/kg/day in one or two doses with meals) because of lower bioavailability.
- Ferrous bisglycinate (FBC; amino-acid chelate): where used clinically, dosing generally aligns with 0.45 mg/kg/day, because of higher absorption [16].
- Vesicular/Encapsulated Liposomal/Sucrosomial Ferric Pyrophosphate: 1.4 mg/kg/day (cap around 29–30 mg/day) has been used in children [17].

#### 3.3.3. Safety and Tolerability

Across compounds, the dominant adverse effects are GI. Ferrous salts predictably cause dose-dependent nausea, abdominal pain, constipation/diarrhea, and metallic taste; liquid forms can stain teeth (mitigated by straw use and dental hygiene) [2,4,18,19]. Chelated, sucrosomial/liposomal, and some ferric preparations show improved GI profiles and palatability in pediatric cohorts, facilitating persistence through the months required to rebuild stores [20]. Age-related differences are nonetheless evident: in infants and young children, poor palatability frequently results in spitting, vomiting, or complete refusal of the medication, effectively precluding adequate dosing, whereas in adults GI adverse effects undermine long-term adherence rather than feasibility of administration. In both age groups, newer formulations—particularly liposomal and sucrosomial iron, which are essentially tasteless and minimize direct mucosal iron exposure—offer a dual advantage of improved tolerability and enhanced adherence, including during prolonged treatment courses. Black stools and stool occult blood positivity can complicate GI evaluation; clinicians should interpret guaiac results in the context of iron therapy. Rare idiosyncratic reactions (rash) are reported with all oral forms; anaphylaxis is largely a concern of parenteral iron, not oral therapy [2,4]. Chronic oral iron misuse can contribute to dysbiosis and constipation; diet fibre and hydration should be emphasized [21].

#### 3.3.4. Pitfalls

The poor palatability of ferrous salts represents a major barrier to effective oral iron therapy, particularly in infants and younger children who often spit out, vomit, or refuse the medication, making administration practically unfeasible. Newer formulations offer two significant advantages: improved acceptability by the child—especially with liposomal and sucrosomial preparations, which are essentially tasteless—and a lower incidence of gastrointestinal side effects, which enhances adherence, even in the context of prolonged treatment courses required for full repletion of iron stores. In addition, it should be noted that liquid drop formulations are generally easier to administer in infants and younger children, as they allow for precise dose adjustment and greater flexibility in delivery, thereby improving acceptability and reducing the likelihood of refusal. A persistent non-response despite adherence should prompt re-evaluation for malabsorption (celiac disease, IBD, Helicobacter pylori infection), ongoing blood loss, or alternative diagnosis; in this scenario, targeted GI evaluation can be revealing and justifies a change in modality (e.g., IV iron), even in pediatrics. Common management pitfalls include stopping iron immediately when hemoglobin normalizes (fosters relapse), under-dosing, and giving iron with meals that markedly inhibit absorption. A recent narrative review catalogues these errors and offers pragmatic fixes for clinicians [21].

#### 3.3.5. Daily vs. Alternate-Day

Emerging evidence suggests that alternate-day dosing of oral iron may represent a rational and better-tolerated strategy in children with iron deficiency or iron-deficiency anemia. The physiological basis lies in the transient hepcidin surge induced by each iron dose, which lasts approximately 24 h and reduces subsequent absorption if daily administration is used. By spacing iron administration every other day, fractional absorption may be enhanced while limiting mucosal exposure and gastrointestinal adverse events [22]. Recent pediatric data, although limited, support this approach. A randomized controlled trial (RCT) including children aged 6 months to 10 years demonstrated that alternate-day iron therapy (in this case oral ferrous fumarate) achieved a greater hemoglobin gain and was associated with fewer gastrointestinal adverse effects compared with twice-daily dosing over 30 days [23]. Similarly, expert consensus recommendations acknowledge alternate-day administration as a reasonable option when gastrointestinal intolerance or adherence issues undermine traditional daily regimens, provided that the total weekly elemental iron dose remains adequate [2]. In practical terms, alternate-day dosing should not result in an overall reduction in elemental iron exposure. For instance, a child requiring 2 mg/kg/day can instead receive 4 mg/kg on alternate days, preserving the cumulative weekly dose. Clinicians should clearly instruct families that the objective is not to lower the overall iron intake but to modify the schedule to improve absorption and tolerability. Although long-term pediatric data remain scarce and current recommendations are still evolving, alternate-day dosing offers an individualized, evidence-based alternative that could balance efficacy and tolerability, and may improve adherence in children who are sensitive to the gastrointestinal side effects of traditional ferrous salts [2,23].

#### 3.3.6. Oral Iron Pharmacology and Absorption: Why Dose, Timing, and Meals Matter

Non-heme iron absorption depends on solubility, redox status, luminal pH, ligand chemistry, DMT1 transport, and the hepcidin–ferroportin axis. Human stable-isotope work demonstrates the following: (1) morning dosing yields higher fractional absorption than afternoon dosing (hepcidin is lower in the morning), (2) coffee and mixed breakfasts depress absorption substantially, and (3) moderate ascorbic acid (~80 mg) enhances absorption, but very high doses add little further benefit. These mechanistic insights align with pediatric practice pearls: dose in the morning, separate from meals and tea/coffee/cocoa; co-ingest with a vitamin-C-containing beverage or fruit when feasible; and beware of calcium-rich foods around the dose. Then, taking ferrous salts on an empty stomach enhances absorption, but increases GI symptoms; real-world practice often individualizes to tolerability. Although these studies were conducted in women, the biological principles plausibly generalize to children and help explain day-to-day variability in pediatric responses. Food inhibitors (phytates, polyphenols), while relevant for dietary iron, may be less consequential for therapeutic iron doses. Prebiotic galacto-/fructo-oligosaccharides may modestly increase absorption from single high-dose supplements in adults; pediatric utility is not yet established [24]. Practical diet measures for toddlers (limit cow milk; emphasize iron-rich foods) remain essential.

### 3.4. Monitoring

After therapy starts, an early check (1–2 weeks) is useful in moderate–severe IDA to confirm Hb trajectory and adherence. On-treatment monitoring should continue to track response and adherence; expect reticulocytosis by ~3–5 days and an Hb increase by ~1–2 weeks. If response remains suboptimal despite confirmed adherence, re-evaluate dose, formulation, administration timing, and dietary/pharmacologic inhibitors; consider hepcidin/Ret-He or inflammatory markers [2,4]. Non-response (after adherence has been confirmed) mandates re-evaluation for occult blood loss, malabsorption (IBD/celiac disease), or alternative diagnosis. In persistent or recurrent IDA with negative celiac screening and no overt bleeding, capsule endoscopy can reveal small-bowel lesions otherwise missed by standard endoscopy, and pediatric series, albeit small, have reported meaningful diagnostic yields, shaping management in refractory cases. Multispecialty guidance further emphasizes that GI investigation is indicated when IDA is severe or unresponsive, always in tandem with appropriate iron replacement rather than waiting for complete etiologic delineation before treatment [20]. In real-world pediatric cohorts, structured monitoring (e.g., AIEOP network) improves detection of non-response and adherence problems and supports timely adjustments [25].

### 3.5. Prophylaxis Versus Therapeutic Supplementation

In the neonatal period, prophylactic oral iron is indicated for preterm and low-birth-weight infants. The American Academy of Pediatrics (AAP) advises elemental iron at 2 mg/kg/day, beginning at 1 month of age and continuing through the first year of life. For infants who remain exclusively breastfed beyond 4 months, the AAP recommends adding 1 mg/kg/day of elemental iron until iron-rich complementary foods are introduced [9]. Prophylactic low-dose iron through fortified formulas or drops reduces the incidence of ID; however, in RCTs of clinic-referred infants and toddlers with established IDA, pharmacologic iron decisively outperforms dietary advice alone for correcting anemia [26]. Comparative prophylaxis trials in infants have evaluated ferrous, ferric, and liposomal preparations, with broadly similar prevention of IDA when dosing and adherence are adequate; formulation choice can be driven by tolerability and adherence rather than presumed large efficacy differences [27]. Historical and contemporary commentaries caution that prophylaxis programmes must be tailored to population risk, nutritional context, and infectious disease ecology; universal supplementation without testing is not a substitute for targeted diagnosis and treatment in clinical care [13].

### 3.6. Oral Iron Formulation-Specific Evidence: Advantages and Drawbacks

#### 3.6.1. Ferrous Salts (Sulfate, Fumarate, Gluconate)

##### Efficacy

Ferrous salts remain the cornerstone of pediatric ID/IDA treatment worldwide and are endorsed as first-line options by contemporary hematology and pediatric consensus groups, primarily on the strength of effectiveness, availability, and low cost [2,5]. Across trials and multicenter cohorts, ferrous sulfate outperformed iron polymaltose/polysaccharide for Hb increase in young children with nutritional IDA [19].

##### Tolerability

GI adverse effects (constipation, abdominal pain, nausea), metallic taste, dental staining, and stool discoloration are frequent, with real-world discontinuation rates that can approach 20–40% in mixed populations, an adherence challenge that is particularly salient in young children averse to bitter liquids [2,18]. Once-daily and alternate-day dosing lower such effects and may improve adherence [2,22,23].

##### Practical

They have a low cost and wide availability; liquid forms facilitate dosing in infants but may stain teeth—counsel on oral hygiene post-dose. Classical pediatric dosing is 2–3 mg/kg/day of elemental Fe^2+^ iron for ID and up to 3–6 mg/kg/day for IDA, delivered once daily or in divided doses (two to three) [2,5]. However, lower dosages of ferrous salts, i.e., 2 mg/kg/day, have been proposed for IDA as still being an efficacious and better-tolerated schedule [15]. Adolescents often use 65–130 mg once daily [2,5]. Practical mitigation strategies include lower elemental iron dosing (no more than 2 mg/kg/day), once-daily morning administration, vitamin C co-ingestion, and alternate-day regimens in those with GI sensitivity [2]. A recent RCT demonstrated that alternate-day ferrous salts therapy is associated with greater hemoglobin gain and fewer gastrointestinal adverse effects compared with twice-daily dosing over 30 days [23].

#### 3.6.2. Ferric Polymaltose/Iron Polymaltose Complex (IPC)

##### Efficacy

Due to the large molecular size and reliance on mucosal dissociation, ferric polymaltose has lower bioavailability than ferrous salts; pediatric meta-analysis and clinical experience show less Hb and ferritin rise compared with ferrous sulfate [28,29].

##### Tolerability

They have a generally better GI tolerability than ferrous salts; they may be an option when ferrous salts are not tolerated, accepting slower correction [18,28].

##### Practical

The IPC role, in practice, hinges on tolerability: in children with significant GI intolerance to ferrous salts, a ferric complex may enable adherence and thus net benefit over time despite slightly slower kinetics of response. They should be considered when adherence is limited by GI symptoms despite optimized ferrous regimens, and dosed at 3–5 mg/kg/day elemental Fe^3+^ in one or two doses with meals [4,5]. Polymaltose is a sugar complex and needs to be dissolved in the gastric fluid to make the iron available in the intestine [5].

#### 3.6.3. Amino-Acid Chelate: Ferrous Bisglycinate

##### Mechanism

Amino-acid chelates are designed to enhance solubility and facilitate uptake via peptide transporters while masking the metallic taste. They have a partly DMT1-independent absorption via peptide transporters (PepT1), potentially mitigating hepcidin effects and dietary inhibitors. Preclinical and in vitro data support enhanced transport vs. ferrous sulfate [16].

##### Efficacy

Pediatric clinical data are heterogeneous. Some studies (including preterm cohorts) suggest non-inferiority at lower elemental doses (approximate 1:4 ratio in some adult settings), with improved GI tolerability and palatability, attributes that can be decisive in pediatrics. Other pediatric multicenter data favoured ferrous salts for Hb increase. Pediatric and perinatal data, including in preterm infants and pregnancy, suggest that ferrous bisglycinate can achieve hematologic endpoints at lower doses with fewer GI symptoms, although high-quality pediatric RCTs remain limited [30].

##### Tolerability

Tolerability is often reported as favourable; they should be considered in children with ferrous salt intolerance, especially when adherence is jeopardized [30].

##### Practical

The Swiss/European pediatric guidance endorses ferrous salts as the first line but acknowledges that alternate formulations (including chelates) are reasonable when intolerance or malabsorption complicate healthcare [5]. Dosing generally aligns with 0.45 mg/kg/day, because of improved tolerability and absorption [16].

#### 3.6.4. Co-Processed Ferrous Bisglycinate with Sodium Alginate (Feralgine™; FBC-A)

##### Mechanism

This technology pairs FBC with sodium alginate via spray-drying to form a gastro-protective, mucoadhesive microenvironment that may improve mucosal tolerance and mask taste [31].

##### Efficacy

Across case series and observational cohorts, including pediatric and GI-disease populations, FBC-A has demonstrated effective rises in hemoglobin and ferritin with high adherence and limited GI adverse effects, including in patients with celiac disease and inflammatory bowel disease (IBD), two groups in whom ferrous salts often fail due to intolerance or poor absorption [31].

##### Tolerability

In IBD, where European Crohn’s and Colitis Organisation (ECCO) consensus frequently favours IV iron in active disease, ref. [31] small observational data suggest FBC-A can be a viable oral option in mucosal disease with acceptable tolerance, but the field still needs adequately powered randomized comparative trials versus ferrous salts and IV iron [31].

##### Practical

Notably, large pediatric multicenter monitoring emphasizes that structured follow-up, irrespective of formulation, predicts success; formulation choice should be integrated into a monitoring framework rather than viewed in isolation. Modelled regimens of 500 mg/day (≈120 mg Fe/day) or 1000 mg/day (≈240 mg Fe/day) have been evaluated in cost-effectiveness analyses. Pediatric dosing is not available and should be individualized if considered [31,32].

#### 3.6.5. Vesicular/Encapsulated Liposomal/Sucrosomial Ferric Pyrophosphate

##### Mechanism

Phospholipid/sucrose ester matrix encapsulates ferric pyrophosphate, facilitating M-cell endocytosis and paracellular uptake, bypassing the divalent metal transporter 1 (DMT1), unlike traditional iron salts; it is proposed to limit mucosal contact and GI irritation [20,33].

##### Efficacy

Prevention: a large infant prophylaxis cohort (retrospective) found ferrous formulations superior to ferric and liposomal iron for preventing ID/IDA at ~1 mg/kg/day, though tolerability was not the focus [27].

Treatment: a recent randomized, double-blind pediatric trial showed liposomal iron improved developmental scores and iron indices vs. placebo in NAID and was effective/tolerable in IDA, with the strongest developmental gains when administered before anemia [34]. In refractory/intolerant adults, sucrosomial iron achieved hematologic correction comparable to IV sodium ferric gluconate at high oral doses, supporting a role for oral sucrosomial iron even where IV iron has been traditional (extrapolation with caution to children). Nevertheless, patient selection (disease activity, CRP, hepcidin) is crucial [33,34]. Doses of 1.4 mg/kg/day (cap around 29–30 mg/day) have been used in children [4,17].

##### Tolerability

In children who struggle with GI adverse effects of ferrous salts, liposomal/sucrosomial ferric pyrophosphate is consistently well-tolerated in trials and real-world pediatric series, with no evidence of therapy-induced intestinal inflammation (calprotectin) and very low reported rates of GI symptoms [20,27,33,34]. However, infant prophylaxis data on tolerability are sparse because major studies focused on efficacy rather than adverse events. Adjunctively, liposomal/sucrosomial ferric pyrophosphate shows excellent palatability, which improves overall therapeutic adherence [34].

##### Practical

They are a reasonable alternative in children with ferrous intolerance or adherence barriers to standard salts, and potentially attractive in NAID where tolerability is paramount; ref. [34] for prophylaxis, ferrous salts remain the first choice where efficacy is critical [20].

#### 3.6.6. Ferric Citrate Hydrate

##### Efficacy

Though most data derive from nephrology and adult populations, cost-effectiveness modelling highlights contexts in which oral ferric citrate can reduce transfusions, ESA requirements, and downstream costs; pediatric-specific evidence is sparse, so any extrapolation should be cautious and individualized [32].

##### Tolerability

As a ferric formulation, its hematologic kinetics may differ from ferrous salts; GI tolerance may be favourable for some children [34].

### 3.7. Synoptical View

Ferrous sulfate typically achieves the fastest hemoglobin rise but suffers from GI intolerance that undermines compliance [2,5,18]. However, lower dosages of ferrous salts have been proposed for IDA as still being an efficacious and better-tolerated schedule [14]. Alternate-day dosing is supported by a recent RCT [23]. Iron polysaccharide/ferric polymaltose often improves GI profile but may yield slower hematologic kinetics [18,28]. Ferrous bisglycinate/co-processed alginate and sucrosomial/liposomal carriers appear to offer a middle path—solid hematologic efficacy at lower elemental doses with consistently better tolerability and palatability in children, including those with GI comorbidities [5,16,20,27,30,31,33,34]. When speed of correction is paramount (e.g., preoperative anemia, severe symptomatic IDA), ferrous sulfate (or IV iron if inflammation is high or oral therapy is failing) is reasonable; when adherence is the bottleneck, a better-tolerated formulation may achieve a larger area under the “absorbed iron × days” curve over months [2]. Finally, formulation choice should be nested within an optimized dosing schedule (lower elemental iron dosing, once-daily morning administration, away from meals, with modest vitamin C co-ingestion; consider alternate-day in sensitive patients) and a monitoring plan that detects non-response by 2–4 weeks and escalates appropriately [2,24]. Dosages, advantages, drawbacks, and other notes for the main pediatric oral iron formulations are summarized in Table 1.

### 3.8. When (and Which) Intravenous Iron?

#### 3.8.1. Indications

Indications include the failure or intolerance of oral therapy, malabsorption (IBD/celiac disease), chronic kidney disease, IRIDA, severe/symptomatic IDA requiring rapid repletion, perioperative optimization, or adherence barriers. Pediatric experience—though smaller than adults—supports good efficacy and safety with modern preparations when administered by experienced teams [2,35].

#### 3.8.2. Agents

High-molecular-weight iron dextran is no longer in clinical use because of its substantial risk of severe anaphylactic reactions. Currently, second-generation intravenous preparations, including low-molecular-weight iron dextran, iron sucrose, and ferric gluconate, are available, with iron sucrose and ferric gluconate being the most widely prescribed owing to their favourable safety profile and minimal association with life-threatening hypersensitivity. A major limitation of these agents is the necessity to fractionate the total replacement dose into multiple infusions. By contrast, third-generation formulations such as ferric carboxymaltose and iron isomaltoside permit the administration of larger amounts of iron in a single session. Among them, ferric carboxymaltose is the only compound currently approved for pediatric patients over 14 years of age, and it offers the additional advantages of requiring smaller cumulative doses and shorter infusion times compared with second-generation products. Dosing and infusion times differ by compound [2,35,36]. Recommended are reported in Table 2.

#### 3.8.3. Dosages

Iron sucrose: 5 mg/kg per dose, administered on alternate days up to 3×/week until the calculated iron deficit is replaced; single-day totals typically ≤300 mg, infused slowly (≥90 min) to limit labile iron and infusion reactions [36].

Ferric gluconate: standard single-infusion dose is 125 mg with a maximum infusion rate ≈ 12.5 mg/min [2]. Because ferric gluconate has a less-stable carbohydrate shell, larger single doses are not permitted, so total iron deficit must be divided over multiple sessions [36]. Total iron deficit can be calculated using an adapted deficit formula:

0.65×weight(kg)×(12−Hbg/dL)×3.3×1.5

The example regimen used is 62.5 mg of ferric gluconate diluted in 250 mL normal saline, continuous IV infusion over 3 h, once per day.

Ferric carboxymaltose (FCM): pediatric dosing used in trials/labelling is 15 mg/kg per infusion (up to 750 mg) on day 0 and day 7 (total 1500 mg) in children ≥ 1 year; phase-2 pediatric data also explored 7.5 mg/kg vs. 15 mg/kg, with larger Hb gains at 15 mg/kg. Weight/Hb-stratified tables (e.g., <35 kg vs. ≥35 kg) are provided in pediatric reviews, offering practical cut-offs for single-visit dosing [36].

Iron isomaltoside/ferric derisomaltose (IIM): planned single doses 10 mg/kg or 20 mg/kg (max 1000 mg) in children < 18 years (ID/IDA intolerant/unresponsive to oral iron); dose selection was informed by adult safety and pharmacokinetics [36].

#### 3.8.4. Safety

Acute serious hypersensitivity is rare with newer agents; however, hypophosphatemia, particularly with FCM, has been reported and warrants awareness and targeted monitoring in repeat/high-dose courses. It should also be noted that, in patients undergoing iron therapy, the risk of developing iron overload, also burdened by a potential pro-infectious effect, must always be monitored [2]. Generally, pediatric narrative reviews conclude IV iron appears safe and achieves faster correction; third-generation products allow full repletion in one or fewer infusions [33,35,36].

#### 3.8.5. Transfusion

Transfusions are reserved for hemodynamic compromise or very severe anemia with symptoms; they are paired with iron repletion afterwards [2].

### 3.9. Practical Pediatric Algorithm (Figure 2, Table 3)

1 Confirm ID/IDA with CBC, ferritin ± TSAT; consider Ret-He and sTfR if inflammation is present [2].
2 If there are the following conditions:
  - Age between 6 months and 2 years
  - Prematurity
  - Puberty
  - Blood donors
  - Pregnancy
  - Gastro-intestinal diseases

Start iron therapy

In all other circumstances, investigate for ID/IDA etiology (see Figure 1), treat the cause and start iron therapy.

3. Start oral ferrous salt (ferrous sulfate, fumarate, gluconate) at 2 mg/kg/day elemental iron for ID, once daily in the morning (preferred way); dosing of 2 mg/kg/day has been proposed for IDA as a still efficacious and better-tolerated schedule [15]. Provide clear adherence and diet counselling [2].
  - Recommend lower elemental iron dosing, once-daily morning administration, away from meals; avoid tea/coffee/cocoa (inhibitors) around dosing; pair with modest vitamin C (fruit/juice) rather than very high pharmacologic doses. Provide age-tailored diet guidance (iron-rich foods, limit cow milk) [18].
  - If prior GI intolerance, poor palatability, or malabsorption risk: consider ferrous bisglycinate or sucrosomial/liposomal iron; start at the lower end of dosing and titrate [2,20,30,33].
  - If active IBD with moderate–severe anemia or high CRP/hepcidin: consider early IV iron per gastroenterology/hematology guidance [37]. Oral options (sucrosomial iron) can be trialled in mild/quiescent disease with close monitoring [20,33].

4. Early check (1–2 weeks) in moderate–severe IDA to verify Hb trajectory and adherence; if Hb ↑ < 1 g/dL or intolerance:
  - Assess adherence, dosing, timing; for GI sensitivity or adherence problems, consider alternate-day dosing without reducing total weekly elemental iron too much [5,17,22,23].
  - Switch formulation if needed: bisglycinate or liposomal for tolerability; accept possibly slower Hb gain vs. ferrous salts [5,16,30,34].

5. Monitor during therapy to assess response and adherence; expect reticulocytosis by ~1 week, Hb rise by ~2–4 weeks [4,25]. If inadequate response and adherence is confirmed, reassess dose, formulation, administration timing, inhibitors, and consider hepcidin/Ret-He or inflammatory markers; in case of non-response after verified adherence, re-evaluate for malabsorption (celiac serology, Helicobacter pylori research in stool), and occult bleeding (including capsule endoscopy if indicated) [2,4,7,20]. consider IV iron [35,36].
6. Continue ≥3 months after Hb normalization to replete stores; verify ferritin on a well day; microcytosis and ferritin lag behind Hb normalization [2,25].

**Figure 2:**
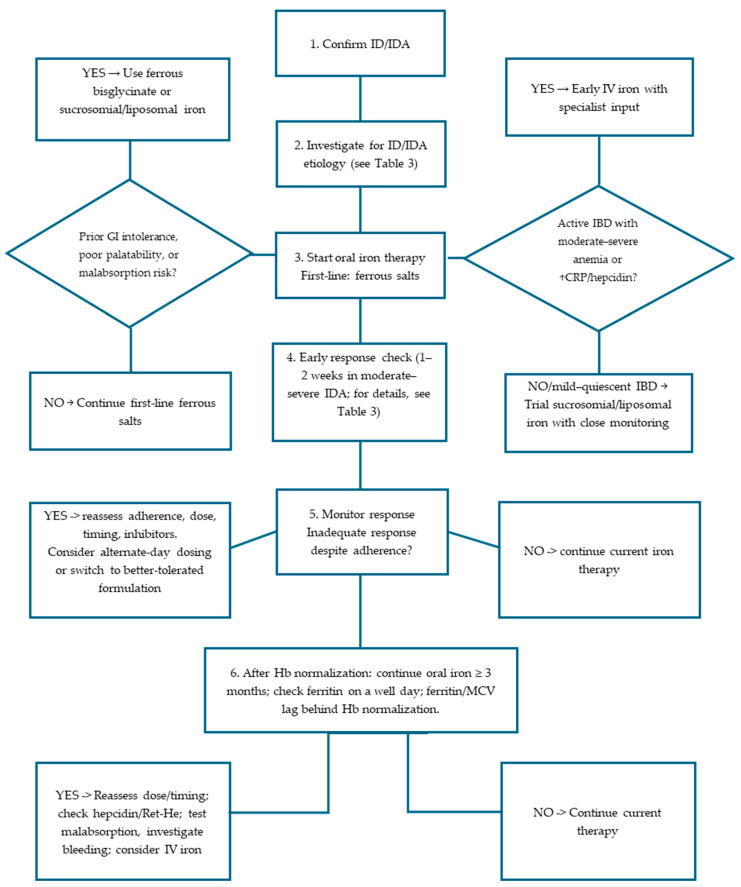
Practical pediatric flowchart for ID/IDA treatment. Abbreviations: ID = iron deficiency; IDA = iron-deficiency anemia; TSAT = transferrin saturation; CRP = C-reactive protein; Ret-He/CHr = reticulocyte hemoglobin content; sTfR = soluble transferrin receptor; FBC-A = co-processed ferrous bisglycinate with alginate; IBD = inflammatory bowel disease.

**Table 3:** ID/IDA diagnosis, etiology, treatment, and monitoring. Abbreviations: ID = iron deficiency; IDA = iron-deficiency anemia; TSAT = transferrin saturation; Hb = hemoglobin; Ret-He/CHr = reticulocyte hemoglobin content; sTfR = soluble transferrin receptor; FBC-A = co-processed ferrous bisglycinate with alginate; GI = gastrointestinal; IBD = inflammatory bowel disease; IV = intravenous; EGD-/RC-scopy = esophagogastroduodeno-/rectocolon-scopy; CE = capsule endoscopy.

Step	Key Action	Details/Notes
1. Confirm ID/IDA	Diagnostic tests	- CBC, ferritin ± TSAT - If inflammation: rely on TSAT, add Ret-He and/or sTfR, if possible (these laboratory parameters are not widely available, remain poorly standardized, and are associated with considerable costs; therefore, they cannot be recommended for routine use in everyday clinical practice yet).
2. Investigate for ID/IDA etiology	If there are any of the following: Age between 6 months and 2 years Prematurity Puberty Blood donors Pregnancy Gastro-intestinal diseases Go to step 3, otherwise search for the underlying condition, and treat the cause, along with iron therapy	Common etiologies: - GI blood loss/IBD: check for occult blood in stool; evaluate EGD- and/or RC-scopy, or CE - Celiac disease: investigate for appropriate serology; if necessary, perform duodenal biopsy - *Helicobacter pylori* infection: research *Helicobacter pylori* in stool
3. Start oral iron therapy	First-line iron	- Ferrous sulfate, fumarate, or gluconate - Dosing: 2 mg/kg/day Prefer once-daily morning dose
	Counselling	- Provide clear guidance on adherence - Educate on dietary factors affecting iron (enhancers/inhibitors)
	If prior GI intolerance, poor palatability, or malabsorption risk	- Use the following: Ferrous bisglycinate Sucrosomial/liposomal iron
	If active IBD with moderate–severe anemia or ↑ CRP/hepcidin	- Early IV iron per specialist input - In mild/quiescent IBD: trial oral sucrosomial iron with monitoring
4. Early response check	At 1–2 weeks in moderate–severe IDA	If Hb increase < 1 g/dL or intolerance: - Assess adherence, dose, timing, dietary inhibitors - For GI sensitivity: consider alternate-day dosing (keep weekly dose stable) - Switch formulation if needed for tolerability (bisglycinate/liposomal); slower response acceptable
5. Monitor therapy and evaluate non-response	Expected timeline	- Reticulocytosis ~1 week - Hb increase in 2–4 weeks
	If poor response despite adherence	- Reassess dose, formulation, administration timing, dietary inhibitors - Check: hepcidin, Ret-He, inflammation markers - Investigate malabsorption: celiac disease serology, *Helicobacter pylori* research in stool - Investigate blood loss: occult blood in stool, EGD- and/or RC-scopy, capsule endoscopy if needed - Consider IV iron if oral therapy fails
6. Continue treatment after Hb recovery	Iron store repletion	- Continue iron for ≥3 months after Hb normalization - Check ferritin on a well day - Note: ferritin and MCV may lag behind Hb normalization

### 3.10. Special Populations

#### 3.10.1. Infants with Dietary Risks

Therapeutic iron is superior to diet alone for IDA correction; dietary measures remain essential to prevent recurrence. Practical family-level guidance (heme sources, fortification, vitamin C pairings, avoidance of tea/cocoa at dosing) is impactful [21]. When prophylaxis is indicated, infant studies comparing ferrous, ferric, and liposomal formulas suggest broadly similar anemia prevention if adherence is high; palatability may steer formulation choice [2,18,19].

#### 3.10.2. Non-Anemic Iron Deficiency (NAID)

Treat when symptomatic or high-risk; pediatric RCT data support developmental benefits of targeted liposomal iron in NAID [15].

#### 3.10.3. IBD/Celiac Disease/Malabsorption

In IBD, active mucosal inflammation increases epithelial permeability and sensitivity to luminal iron. Ferrous salts can exacerbate abdominal pain, diarrhea, and nausea and may increase oxidative stress in the inflamed mucosa. Inflammation-driven hepcidin elevation reduces absorption, leaving more unabsorbed iron in the lumen and worsening GI symptoms. For this reason, intolerance to conventional oral iron is frequent in active IBD. Guidelines frequently privilege IV iron in active disease with moderate-to-severe anemia [35,36]. Still, clinical series suggest that FBC-A and sucrosomial iron can succeed in selected pediatric IBD when disease is quiescent or mild, enabling oral therapy where ferrous salts fail [31,33,37].

Untreated or newly diagnosed celiac disease is characterized by villous atrophy and impaired absorptive capacity. Oral iron, especially ferrous salts, often causes bloating, abdominal pain, and diarrhea and may be poorly absorbed. Even after the initiation of a gluten-free diet, mucosal recovery can take months, during which GI intolerance to iron remains common. Celiac disease is a frequent cause of refractory IDA; co-processed bisglycinate/alginate shows favourable absorption even early in the gluten-free diet, with case-series documenting the normalization of Hb and ferritin and excellent tolerance [30,31,33]. The pursuit of etiology of ID/IDA should proceed in parallel with iron repletion [7,20].

#### 3.10.4. Adolescents (e.g., Heavy Menstrual Bleeding)

The same principles apply; ensure adequate dosing and adherence; consider IV iron if rapid repletion is needed or intolerance occurs. Ferrous salts are effective, but GI tolerance and adherence often determine success; alternate-day schedules and better-tolerated formulations (bisglycinate, sucrosomial) can help sustain multi-month therapy [2].

#### 3.10.5. Chronic Infections and Inflammatory States

Recurrent infections, obesity-related inflammation, or chronic systemic inflammatory conditions raise hepcidin levels, reducing iron absorption. The resulting increase in unabsorbed luminal iron contributes to nausea, abdominal pain, and dysbiosis, negatively affecting tolerance. Pediatricians can mitigate poor GI tolerance through a stepwise, individualized approach. Optimizing dosing and schedule (lowest effective dose, e.g., 2 mg/kg/day in many cases; once-daily morning dosing) and/or modifying the formulation (switch from ferrous salts to better-tolerated formulations such as ferrous bisglycinate, or sucrosomial/liposomal iron, which reduce direct mucosal exposure and improve palatability; liquid or drop formulations, favouring tasteless or minimally flavoured products) allows the subject to maintain effective iron repletion while minimizing adverse effects and treatment failure [2,5].

### 3.11. Cost-Effectiveness and Health-System Considerations

Direct pediatric pharmacoeconomic data are scarce. Adult analyses indicate that avoiding hospital-based IV administration with well-tolerated, effective oral options (including sucrosomial/liposomal) can be cost-effective when they obviate infusion-related costs. In routine pediatric practice, the most cost-effective regimen is the one a child will actually take for the full course: a slightly more expensive, better-tolerated formulation can be economically rational if it avoids repeated visits, labs, and therapy failures. Nonetheless, IV iron remains cost-effective where rapid repletion avoids complications [21,32,37].

### 3.12. Gaps and Future Directions

This work has some limitations that should be acknowledged. First, it is a narrative review, and therefore does not follow a systematic methodology with predefined inclusion criteria, formal risk-of-bias assessment, or quantitative synthesis; as such, the conclusions rely on a critical interpretation of heterogeneous evidence rather than pooled effect estimates. Second, high-quality pediatric randomized controlled trials directly comparing different oral iron formulations are limited, and much of the available evidence is derived from small trials, observational studies, or extrapolation from adult data, which may not fully capture age-specific differences in absorption, tolerability, and adherence.

Third, formulation-specific outcomes such as palatability, acceptability, and real-world adherence are inconsistently measured and rarely standardized, limiting cross-study comparisons despite their central importance in pediatric practice. Fourth, emerging biomarkers such as hepcidin, Ret-He, and soluble transferrin receptor, while biologically informative, remain poorly standardized, variably available, and costly, restricting their routine use and limiting the generalizability of biomarker-guided strategies discussed in this review.

Finally, the evidence base is influenced by contextual heterogeneity, including differences in nutritional background, infection burden, health-system resources, and socioeconomic factors across study settings, which may affect both treatment response and safety profiles.

The pediatric field would benefit from the following: (1) adequately powered RCTs comparing ferrous sulfate with bisglycinate/alginate and sucrosomial iron on both hematologic endpoints and patient-centred outcomes (adherence, QoL, neurodevelopment); (2) hepcidin-guided dosing trials (daily vs. alternate-day) in children; (3) head-to-head tolerability and palatability studies using child-validated scales; (4) standardized pediatric safety surveillance for IV iron (including hypophosphatemia risk stratification); (5) trials stratified by inflammation (IBD/celiac disease) and by baseline hepcidin/CRP; and (6) rigorous cost-effectiveness studies that account for adherence failures and rescue IV iron.

## 4. Discussion

ID and IDA remain the most prevalent nutritional disorders in childhood, with substantial implications for growth, neurocognitive development, and overall health. In children, oral iron remains the keystone of ID/IDA management. For most children with ID/IDA, ferrous salts (especially ferrous sulfate) remain first-line owing to consistent efficacy, low cost, and broad guideline endorsement. Dose optimization comprehends lower elemental iron dosing (no more than 2 mg/kg/day of ferrous salts), once-daily morning administration, away from meals and with modest-dose vitamin C. Despite the long-standing role of oral ferrous salts as the first-line treatment, their gastrointestinal intolerance and adherence challenges often limit therapeutic success in pediatric practice [2,5]. Alternate-day dosing is supported by a recent pediatric RCT for improving Hb response and GI tolerability. Although long-term pediatric data are still limited, alternate-day dosing offers an evidence-based alternative that balances efficacy and tolerability, and may improve adherence in children who are sensitive to GI side effects of traditional ferrous salts [2,23]. Recent advances in alternative oral formulations, including ferric complexes, amino-acid chelates, and encapsulated preparations such as sucrosomial and liposomal ferric pyrophosphate, offer meaningful improvements in tolerability, palatability, and adherence, often at lower elemental doses, while maintaining hematologic efficacy in many settings. Pediatric data, though still more limited than adult evidence due to small sample sizes and currently short follow-up, support the clinical value of these newer compounds, particularly in children with intolerance to traditional salts, malabsorption, or comorbidities such as inflammatory bowel disease or celiac disease [2,5,16,17,30,33].

The choice of therapy should therefore be individualized, balancing the need for rapid correction of anemia against long-term adherence, tolerability, and safety. Ferrous sulfate continues to provide the fastest hematologic response and remains the preferred option for prophylaxis in infants at high risk, [2], whereas newer encapsulated or chelated formulations may achieve better persistence and quality of life in children for whom adherence is the main barrier [16,17,30,33]. Intravenous iron, while safe and effective with modern preparations, should be reserved for selected cases of severe disease, malabsorption, or failed oral therapy [35,36].

Looking forward, adequately powered pediatric randomized controlled trials are needed to compare formulations and administration methods head-to-head, integrate biomarkers such as hepcidin into dosing strategies, and assess broader outcomes including neurodevelopment, quality of life, and cost-effectiveness. Until such data are unavailable, the optimal management of pediatric ID and IDA will depend on a pragmatic, patient-centred approach, matching compound, dose, and schedule to age, phenotype, comorbidity, and adherence constraints, close monitoring and prompt adaptation of formulation, dosing schedule, or route of administration; when intolerance or non-response occurs, this approach should be integrated [2,5]. This tailored strategy, supported by structured follow-up and family education, offers the best prospect to reduce the global burden of iron deficiency in children.

## Figures and Tables

**Table 1:** Dosages, advantages, drawbacks, and other notes for the main pediatric oral iron formulations.

Iron Formulation	Recommended Dosage	Advantages	Drawbacks	Notes
Ferrous salts	2–6 mg/kg/day in 1–3 doses	Standard treatment Good absorption Low cost	Frequent gastro-intestinal side effects Metallic taste Dental staining Stool discoloration	Dosage of 2 mg/kg/day has been proposed as still being an efficacious and better-tolerated schedule; prefer once-daily morning dose; alternate-day dosing is supported by a recent RCT [23].
Ferric polymaltose/Iron polymaltose complex	3–5 mg/kg/day in 1–2 doses	Better gastro-intestinal tolerability than ferrous salts	Less effective than ferrous salts	
Ferrous bysglicinate	0.45 mg/kg/day in 1–2 doses	Good intestinal absorption Limited side effects	Higher cost than ferrous salts	
Liposomal/sucrosomial ferric pyrophosphate	1.4 mg/kg/day in 1–2 doses	Excellent palatability Limited side effects	Possible less prompt response to therapy Less effective in prophylaxis than ferrous salts Higher cost than ferrous salts	Sparse prophylaxis data on tolerability

**Table 2:** Dosages, advantages, drawbacks, and other notes for the main intravenous iron formulations.

Iron Formulation	Recommended Dosage	Advantages	Drawbacks	Notes
Iron sucrose	5 mg/kg/dose, on alternate days, up to 3×/week (until iron deficit is replaced); single-day dose ≤ 300 mg, infused ≥ 90 min		Hospitalization required Multiple infusions	
Ferric gluconate	Total dose to be calculated based on initial Hb and weight	Effectiveness independent of gastro-intestinal absorption Very low gastro-enteric side effects	Hospitalization required Multiple infusions	
Ferric carboxymaltose (FCM)	15 mg/kg per infusion (up to 750 mg) on day 0 and day 7 (total 1500 mg) in children ≥ 1 year	Effectiveness independent of gastro-intestinal absorption Once-weekly administration	Hospitalization required Higher risk of hypophosphatemia compared to other formulations	Phase-2 pediatric data explored 7.5 mg/kg per infusion dosing
Iron isomaltoside/Ferric derisomaltose (IIM)	10–20 mg/kg (max 1000 mg) in children < 18 years	Effectiveness independent of gastro-intestinal absorption Single administration	Hospitalization required	

## Data Availability

No new data were created or analyzed in this study.
